# Interaction of antihistaminic drugs with human translationally controlled tumor protein (TCTP) as novel approach for differentiation therapy

**DOI:** 10.18632/oncotarget.7605

**Published:** 2016-02-23

**Authors:** Ean-Jeong Seo, Thomas Efferth

**Affiliations:** ^1^ Institute of Pharmacy and Biochemistry, Department of Pharmaceutical Biology, Johannes Gutenberg University, 55128 Mainz, Germany

**Keywords:** translationally controlled tumor protein (TCTP), antihistaminic compounds, levomepromazine, buclizine, cancer differentiation therapy

## Abstract

Translationally controlled tumor protein (TCTP) represents an exquisite target for cancer differentiation therapy, because it was most strikingly down-regulated in tumor reversion experiments. Since TCTP is identical with the histamine releasing factor, antihistamic drugs may inhibit TCTP. Indeed, antihistaminics, such as promethazine, thioridazine, perphemazine and chlorpromazine reveal antiproliferative effects. The aim of this investigation was to study antihistaminic drugs as new TCTP inhibitors to inhibit tumor growth. Levomepromazine and buclizine showed higher *in silico* binding affinities to TCTP among 12 different antihistaminic compounds including the control drugs, promethazine and hydroxyzine by using Autodock4 and AutodockTools-1.5.7.rc1. Recombinant human *TCTP* was codon-optimized, expressed in *E. coli* and purified by chitin affinity chromatography. For experimental validation of *in silico* data, we applied microscale thermophoresis. Levomepromazine bound with a Kd of 57.2 μM (*p* < 0.01) and buclizine with a Kd of 433μM (*p* < 0.01) to recombinant TCTP. Both drugs inhibited MCF-7 breast cancer cell growth in resazurin assays. TCTP expression was down-regulated after treatment with the two drugs. Cell cycle was arrested in the G1 phase without apoptosis as confirmed by the expression of cell cycle and apoptosis-regulating proteins. Annexin V-PI staining and Trypan blue exclusion assay supported that the two drugs are cytostatic rather than cytotoxic. Induction of differentiation with two drugs was detected by the increased appearance of lipid droplets. In conclusion, levomepromazine and buclizine inhibited cancer cell growth by binding to TCTP and induction of cell differentiation. These compounds may serve as lead compounds for cancer differentiation therapy.

## INTRODUCTION

Cancer is one of the leading causes of death all over the world. Traditionally, surgery, chemotherapy and radiotherapy are main treatment options and cytotoxic drugs are indispensable in the armory to destroy tumor cells [1]. However, many cytotoxic agents reveal side effects such as bone marrow suppression, gastrointestinal tract lesions, hair loss, nausea *etc.*, because these agents are active on both, proliferating, malignant tumor and healthy, normal cells. Therefore, these drugs induce cell death not only in tumors, but also in normal cells [2, 3]. Since cytotoxic drugs lack sufficient tumor selectivity, they frequently cannot cure patients due to non-tolerable high side effects that prevent the application of drug doses high enough to sustainably kill all cells of a tumor.

Another concept is differentiation therapy, which aims at re-activation of endogenous differentiation programs in cancer cells with subsequent cellular maturation and loss of the aggressive tumor phenotype [4]. For instance, retinoids play a fundamental role in chemoprevention of carcinogenesis and in differentiation therapy [5]. Treatment of osteosarcoma and chondrosarcoma cells with all-trans retinoic acid (ATRA) inhibited tumor growth in a reversible manner [6, 7]. Besides, ATRA hypophosphorylated RARα inhibiting cellular proliferation and inducing osteoblastic differentiation [8].

A novel target for differentiation therapy is the translationally controlled tumor protein (TCTP), because it was the most down-regulated gene in tumor reversion experiments [9]. Tumor reversion is a biological process, by which highly tumorigenic cells lose their malignant phenotype [10, 11]. Reversion is regulated by proteins such as seven in absentia homologue (SIAH1), presenilin 1 (PS1), tumor suppressor activated pathway (TSAP), and TCTP [10]. Inhibition of TCTP expression increased the number of revertant cells, which regained sensitivity to contact inhibition and decreased tumor-forming capability [9, 11].

TCTP has also been named histamine releasing factor (HRF), fortilin, P21, P23, TPT-1 and Q23 [12–14]. It is a highly conserved pro-survival factor in eukaryotes and is ubiquitously expressed in various tissues and cells [15]. Besides, TCTP is a multifunctional protein, which plays important roles in numerous cell physiological events, such as immune response, cell proliferation, tumorigenicity, and cell death. Its overexpression in cancer patients speaks for its possible clinical relevance [15, 16].

Thus, TCTP represents an exquisite target for anti-cancer differentiation therapy. The antihistaminics promethazine and hydroxyzine inhibit TCTP [9, 16] giving a first hint, that antihistaminic drugs may be a suitable class of TCTP inhibitors. Therefore, we systematically investigated a panel of antihistamic compounds for their interaction with TCTP as new inhibitors of human TCTP and tumor growth.

## RESULTS

### Molecular docking of 12 antihistaminic compounds to human TCTP

**Figure 1:**
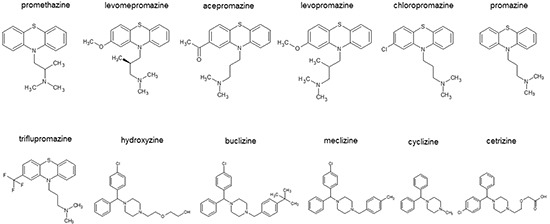
Chemical structures of antihistaminic compounds investigated by *in silico* molecular docking

Initially, we performed blind molecular dockings with each 100 runs to predict binding energies of 10 antihistaminic compounds and two control drugs (promethazine and hydroxyzine) to human TCTP (Figure 1 and Table 1). Levomepromazine and buclizine showed the highest binding affinities and were therefore selected to study the interaction to human TCTP in more detail.

**Table 1:** *In silico* blind molecular docking of promethazine and hydroxyzine and their related substances to human TCTP. Dockings were performed with 100 runs for each compound

Compounds	Lowest energy of docking (kcal/mol)	Mean binding energy(kcal/mol)	Residues involved hydrogen bond interaction with the ligand	Residues involved in hydrophobic interaction with ligand	pKi (μM)
**Promethazine (control drug)**	−6.39	−5.99	Asp 44, Thr 65	Thr 39, Gly 41, Ile 43, Asp 44, Glu 63, Ser 64, Thr 65	20.73
**Levomepromazine**	−7.10	−6.35	Ser 64	Thr 39, Gly 41, Ile 43, Asp 44, Ile 48, Glu 63, Ser 64, Thr 65	6.26
**Acepromazine**	−6.88	−6.39	Ser 64	Thr 39, Ile 43, Asp 44, Ile 48, Thr 62, Glu 63, Ser 64, Thr 65	9.05
**Levopromazine**	−6.88	−6.19	Ser 64	Thr 39, Asp 44, Ile 48, Glu 63, Ser 64, Thr 65	8.99
**Chloropromazine**	−6.49	−6.21	-	Thr 39, Glu 40, Ile 43, Asp 44, Glu 63, Ser 64, Thr 65	17.55
**Promazine**	−6.25	−5.88	-	Thr 39, Glu 40, Asn 42, Asp 44, Ile 48, Glu 63, Thr 65	26.11
**Triflupromazine**	−5.93	−5.58	Thr 65	Thr 39, Ile 43, Asp 44, Ile 48, Thr 62, Glu 63, Ser 64, Thr 65	45.14
**Hydroxyzine (control drug)**	−7.87	−6.73	Thr 62, Glu 63	Thr 39, Glu 40, Gly 41, Asn 42, Ile 43, Asp 44, Ile 48, Glu 60, Gly 61, Th62, Glu 63, Thr 65	1.69
**Buclizine**	−8.35	−7.77	Glu 63	Thr 39, Glu 60, Gly 61, Thr 62, Glu 63, Ser 64, Thr 65, Val 66	0.76
**Meclizine**	−8.09	−7.53	Glu 63	Glu 40, Asn 42, Ile 43, Asp 44, Glu 60, Gly 61, Glu 63, Thr 65	1.17
**Cyclizine**	−7.58	−7.32	Glu 63	Thr 39, Glu 40, Asn 42, Ile 43, Asp 44, Ile 48, Glu 63, Thr 65	2.78
**Cetrizine**	−6.20	−6.20	Lys 171	Met 1, Typ 4, Asp 16, Typ 18, Lys 19, Ile 20, Glu 22, Leu 29, Lys 171	28.33

**Figure 2:**
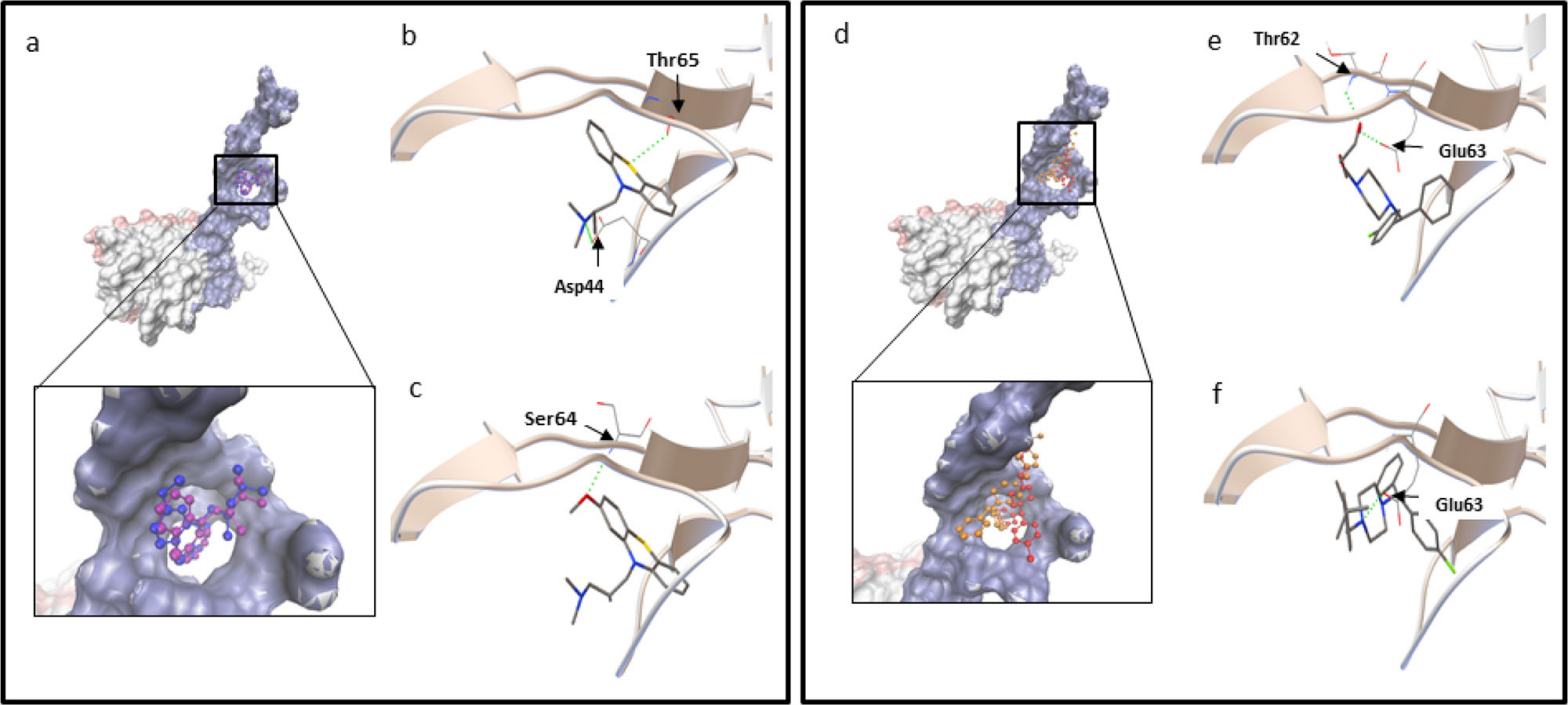
(a-c) Molecular docking of promethazine and levomepromazine **a.** Docking of promethazine (in purple) and levomepromazine (in blue) into the binding site of human TCTP (PDB code: 2HR9 in surface representation, blue surface represents Ca^2+^ binding site, TCTP residues: 1-70 [32]; pink surface represents p53 binding site, TCTP residues: 70-119 [52] and grey surface represents Bcl-xL binding site, TCTP residues: 20-27 [51]). Levomepromazine (blue) occupied the same binding site as promethazine (purple). Docked structure of promethazine **b.** and levomepromazine **c.** in the human TCTP binding pocket. **d-f.** Molecular docking of hydroxyzine and buclizine. (d) Docking of hydroxyzine (in red) and buclizine (in orange) into the binding site of human TCTP (PDB code: 2HR9 in blue surface representation (Ca^2+^ binding site)). Buclizine (orange) occupied the same binding site as hydroxyzine (red). Docked structure of hydroxyzine (e) and buclizine (f) in the human TCTP binding pocket. The residues involved in hydrogen bond interaction are labeled and hydrogen bonds are shown as green dots.

Afterwards, defined molecular dockings of promethazine, hydroxyzine, levomepromazine and buclizine were performed three times with a grid laid around human TCTP residues found by blind docking (Table 2). Levomepromazine (blind docking: −7.10 kcal/mol, defined docking: −8.02 kcal/mol) showed much lower binding energy to human TCTP than promethazine (blind docking: −6.39 kcal/mol, defined docking: −6.82 kcal/mol) (Tables 1 and 2). Moreover, buclizine (blind docking: −8.35 kcal/mol, defined docking: −9.49 kcal/mol) revealed higher affinity to human TCTP than hydroxyzine (blind docking: −7.87 kcal/mol, defined docking: −8.90 kcal/mol) by both blind and defined docking approaches (Tables 1 and 2). Besides, levomepromazine and buclizine bound to the same sites as promethazine and hydroxyzine, respectively (Figure 2).

**Table 2:** Defined molecular docking to TCTP of compounds selected by blind dockings (Table 1). Dockings were performed three times with 250 number per run with TCTP residues found by blind docking

Compounds	Lowest energy of docking (kcal/mol)	Mean binding energy(kcal/mol)	Residues involved hydrogen bond interaction with the ligand	Residues involved in hydrophobic interaction with ligand	pKi (μM)
**Promethazine (control drug)**	−6.82±<0.00	−6.82±<0.00	Asp 44, Thr 65	Thr 39, Gly 41, Ile 43, Asp 44, Glu 63, Ser 64, Thr 65	9.98±<0.00
**Levomepromazine**	−8.02±0.01	−7.76±<0.00	Ser 64	Thr 39, Gly 41, Ile 43, Asp 44, Glu 63, Ser 64, Thr 65	1.32±0.02
**Hydroxyzine (control drug)**	−8.90±0.06	−8.27±0.02	Asn 51	Gly 41, Asn 42, Ile 43, Asp 44, Ile 48, Asn 51, Glu 60, Gly 61, Th62, Glu 63, Thr 65	0.30±0.03
**Buclizine**	−9.49±0.02	−9.01±0.03	-	Thr 39, Gly 41, Asn 42, Ile 43, Asp 44, Ile 48, Gly 50, Asn 51, Glu 60, Gly 61, Thr 62, Glu 63, Thr 65	0.11±<0.00

### Codon optimization of the human *TCTP* gene for expression in *E. coli*

**Figure 3:**
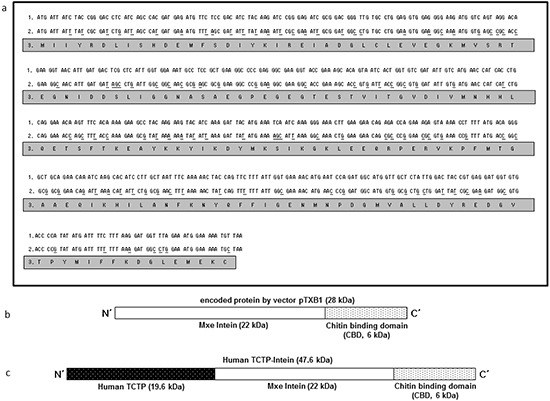
**a.** Comparison of human TCTP sequences. Lane 1: original sequences of human *TCTP* (GenBank accession no. X16064.1); Lane 2: optimized sequences of human *seTCTP* (EMBL LN881713); Lane 3: sequences of corresponding amino acids of human TCTP. **b. c.** Schematic composition of intein-chitin binding domain (b) and human TCTP-intein (c).

In order to study the interaction of antihistaminic drugs and human TCTP experimentally, we expressed human TCTP in *E. coli* in a heterologous manner. Differences in codon usage between species can affect quantity and quality of recombinant protein expression. Therefore, human *TCTP* was screened for the presence of rare codons (GenBank accession no. X16064.1). Human TCTP consists of 172 amino acids. Of them, 28 amino acids (16%) are encoded by rarely used codons in *E. coli* (Figure 3a). To avoid potential problems of rare codons for human *TCTP* expression in *E. coli*, a corresponding new gene was designed, termed human *seTCTP* (EMBL LN881713) (Figure 3 lane 2). Here, rare codons were replaced by those frequently used in *E. coli* according to common codon usage of *E. coli* (http://www.kazusa.or.jp/codon/) (Figure 3a). This synthetic gene was synthesized by Eurofins MWG Operon (Ebersberg, Germany) according to the DNA sequences designed by us.

### Construction of recombinant plasmids for human *seTCTP* expression

Plasmid vector pTXB1 (NEB, Figure 3b) encodes an Intein-tag and allows gene expression under the control of the T7 promoter. This plasmid was used to construct a recombinant plasmid coding for a fusion protein consisting of a C-terminal Intein-tag and human TCTP. The synthetic human *seTCTP* was ligated with the cleaved pTXB1 to obtain the expression plasmid pTCTP01. The gene was under the control of the T7 promoter and the resulting human *seTCTP* contained a C-terminal Intein-tag (human *TCTP*-Intein) (Figure 3c).

### Expression of human *seTCTP* in *E. coli* K12 ER2566 harboring plasmid pTCTP01, affinity purification and on-column cleavage of human TCTP

**Figure 4:**
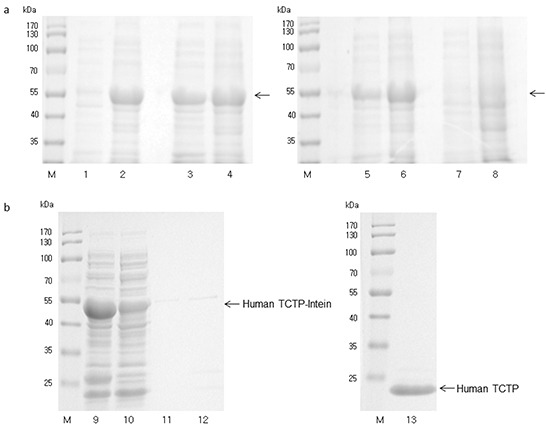
**a.** SDS-PAGE analysis of solubility of human TCTP-intein protein (47 kDa) from ERTXB101 after expression with 0.1 mM IPTG and various temperatures (Samples were adjusted to the number of bacterial cells (3.84 × 10^9^ cfu). Arrows indicate the expressed human TCTP-intein (47 kDa). M: Protein ladder, (Thermo Scientific), Lane 1: 37°C, 3 h, soluble proteins, Lane 2: 37°C, 3 h, insoluble proteins, Lane 3: 27°C, 5 h, soluble proteins, Lane 4: 27°C, 5 h, insoluble proteins, Lane 5: 18°C, 16 h, soluble proteins, Lane 6: 18°C, 16 h, insoluble proteins, Lane 7: 18°C, 16 h, non-induced soluble proteins, Lane 8: 18°C, 16 h, non-induced insoluble proteins. **b.** SDS-PAGE analysis of affinity purification of ERTXB101 by chitin column after induction of protein expression at 27°C for 5 h with washing buffers in the presence of 1 mM EDTA, M: Protein ladder (Thermo Scientific), Lane 9: loading sample, Lane 10: flow through from chitin column, Lane 11: wash, Lane 12: DTT flush to distribute it evenly throughout the column, Lane 13: elution of human TCTP (19 kDa) after stopping column flow and inducing cleavage reactions.

The expressed human TCTP was solvable after induction at various temperatures (37°C, 27°C and 18°C) and 0.1 mM isopropyl ß-D-thiogalactopyranoside (IPTG). Afterwards, induced cultures were centrifuged and the pellets were dissolved in cell lysis buffer. Bacterial cell numbers were monitored by OD values and the bacterial cell numbers were adjusted to 1.28 × 10^10^ cfu/mL in cell lysis buffer. Then, cell lysis was performed by sonication. After centrifugation, the supernatant contained soluble proteins. Insoluble proteins were also gathered by dissolving pellet in 8 M urea cell lysis buffer after sonication. The highest yield of soluble human TCTP-intein protein was obtained after induction at 27°C (Figure 4a).

Soluble human TCTP-intein protein was purified as described in Material and Methods. SDS-PAGE was performed to analyze affinity purification and on-column cleavage (Figure 4b). Thereby, we obtained purified recombinant human TCTP for further investigation of the interactions between human TCTP and antihistaminic compounds.

### Molecular interaction studies using microscale thermophoresis

**Figure 5:**
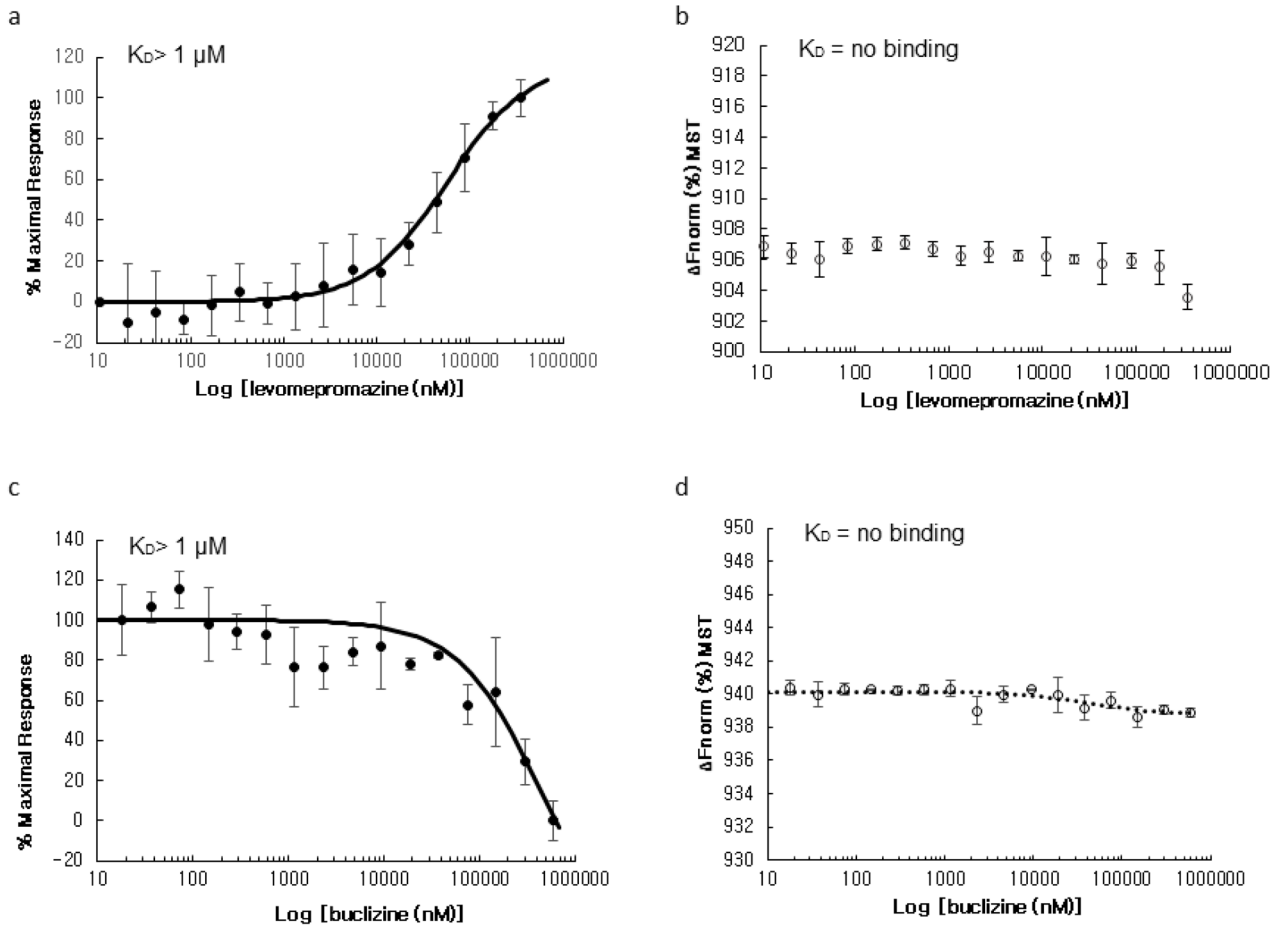
Microscale thermophoresis of the levomepromazine-human TCTP a. and buclizine-human TCTP complexes **c.** and human TCTP alone in assay buffer (negative control) **b.** and in adapted assay buffer including 2% chloroform (negative control) **d.** Values on the Y-axis represent the thermophoretic shift of labeled human TCTP. Each experiment was performed at least three times and values are represented as mean ± SD.

We used microscale thermophoresis for analysis and quantification of direct interactions of human TCTP with levomepromazine and buclizine (Figure 5). This method allows the determination of binding affinities between fluorescently labeled target proteins and non-labeled compounds. We titrated labeled human TCTP with increasing concentrations of levomepromazine or buclizine. The levomepromazine concentration-dependent increase gave an apparent equilibrium binding constant of 57.2 ± 6.49 μM (*p* < 0.01) for levomepromazine. Buclizine showed a concentration-dependent decrease yielding a binding affinity of 433 ± 47.1 μM (*p* < 0.01). These results provided evidence for direct binding of human TCTP to both levomepromazine and buclizine.

### Growth inhibition of MCF-7 cells by levomepromazine and buclizine

**Figure 6:**
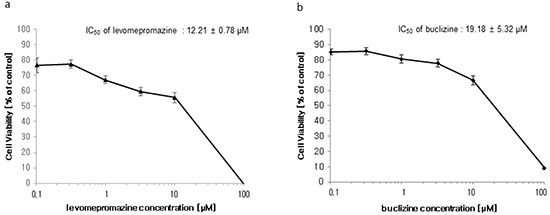
Growth inhibition of MCF-7 cells by levomepromazine **a. and buclizine b.** Cells were treated with different concentrations or vehicle control for 72 h and subsequently resazurin reduction assays were performed. Viability of cells was represented by mean ± SD of three independent experiments and was expressed as percentage survival of control.

The growth inhibitory effects of levomepromazine and buclizine were tested by using MCF-7 cells. Treatment of cells with different concentrations for 72 h showed that levomepromazine (IC_50_: 12.21 ± 0.78 μM) and buclizine (IC_50_: 19.18 ± 5.32 μM) revealed considerable growth inhibition (Figure 6).

### TCTP expression of MCF-7 cells after levomepromazine or buclizine treatment

**Figure 7:**
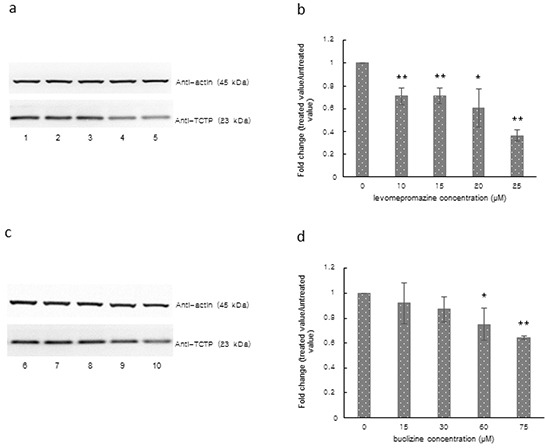
TCTP expression after treatment with levomepromazine **a, b. or buclizine c, d. for 72 h as analyzed by Western blot,** Lane 1: solvent (H_2_O), Lane 2: 10 μM, Lane 3: 15 μM, Lane 4: 20 μM, Lane 5: 25 μM, Lane 6: solvent (DMSO), Lane 7: 15 μM, Lane 8: 30 μM, Lane 9: 60 μM and Lane 10: 75 μM (Significantly different according to Student's *t*-test, * 0.01 < *P* ≤ 0.05, **P ≤ 0.01), Quantification of TCTP (b and d) expression by ImageJ. Western blots were performed three times.

We observed a significantly decreased TCTP expression in MCF-7 cells after treatment with 10-25 μM levomepromazine or 60-75 μM buclizine for 72 h (Figure 7). TCTP expression was decreased by more than 60% by 25 μM levomepromazine (Figure 7a and 7b). and by 40% by 75 μM buclizine (Figure 7c and 7d).

### Cell cycle analysis

**Figure 8:**
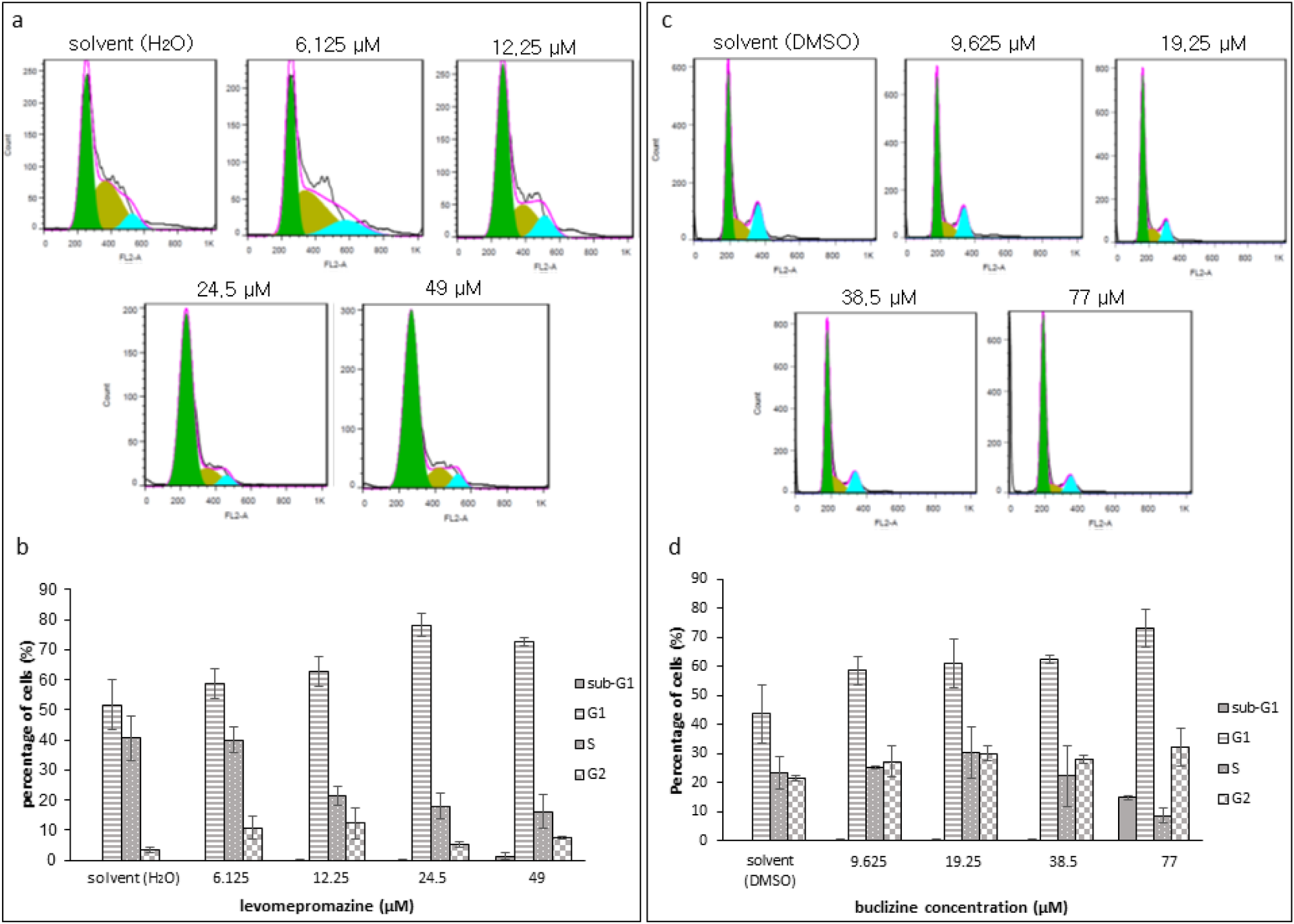
Cell cycle analysis of MCF-7 cells after treatment with levomepromazine (a and b) or buclizine (c and d) for 72 h The graphs are mean values and standard deviations of three independent experiments.

TCTP is essential for the orderly cell cycle transition. However, increased TCTP induces mitotic defects and chromosome miss-segregation in cancer cells [17]. In this study, levomepromazine and buclizine downregulated TCTP expression. Therefore, we investigated the effect of levomepromazine and buclizine on the cell cycle. After incubation for 72 h, levomepromazine and buclizine inhibited cell growth of MCF-7 cells and arrested the cell cycle in the G1 phase in a dose-dependent manner after 72 h (Figure 8). The percentages of cells in the G1 phase increased to 78% and 73%, with 24.5 μM and 49 μM levomepromazine, respectively. Similarly, G1 phase fractions raised to 73% by 77 μM buclizine (Figure 8).

### Assessment of the mode of action of levomepromazine and buclizine toward MCF-7 cells by annexin V-PI staining

**Figure 9:**
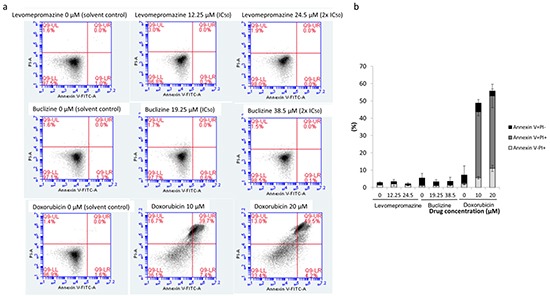
Analysis of cell death in MCF-7 cells induced by levomepromazine, buclizine or doxorubicin **a.** Representative dot plots of flow cytometry analysis after treatment of MCF-7 cells with IC_50_ or 2 × IC_50_ of levomepromazine and buclizine as well as 10 μM or 20 μM doxorubicin for 72 h. **b.** The graph of the means ± SD of three independent experiments. Annexin V-/PI+: late necrosis, annexin V+/PI+: late apoptosis or early necrosis, annexin V+/PI-: early apoptosis.

Cell cycle analysis showed G1 arrest without apoptosis after treatment with levomepromazine and buclizine, we also investigated the action of levompromazine and buclizine by annexin V-PI staining (Figure 9). After treatment of MCF-7 cells with IC_50_ or 2 × IC_50_ concentrations of levomepromazine or buclizine for 72 h, most of cells were alive (annexin V-/PI-), whereas doxorubicin, used as cytotoxic control drug, caused dramatic induction of cell death with more than 40% cells in late apoptosis (annexin V+/PI+) (Figure 9).

### Western blot analysis of cell cycle regulatory proteins after treatment with levomepromazine or buclizine

**Figure 10:**
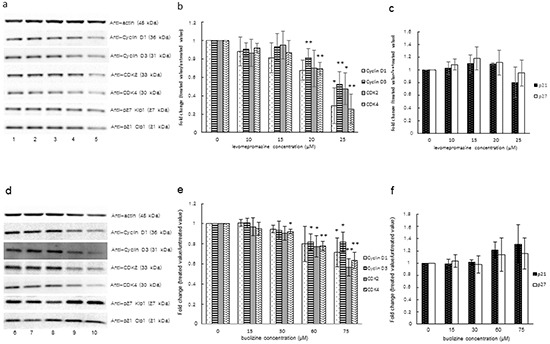
Expression of cyclin D1, cyclin D3, CDK2, CDK4 and cyclin-dependent kinase inhibitors (p21 and p27) after treatment of MCF-7 cells with levomepromazine (a-c) or buclizine (d-f) for 72 h as analyzed by Western blot Lane 1: solvent (H_2_O), Lane 2: 10 μM, Lane 3: 15 μM, Lane 4: 20 μM, Lane 5: 25 μM, Lane 6: solvent (DMSO), Lane 7: 15 μM, Lane 8: 30 μM, Lane 9: 60 μM and Lane 10: 75 μM (Significantly different according to Student's *t*-test, * 0.01 < *P* ≤ 0.05, **P ≤ 0.01), Quantification of protein expression by ImageJ. Western blot were performed three times.

Since we observed G1 arrest after treatment with levomepromazine or buclizine, we investigated the effect of two drugs on cell cycle regulatory proteins, *i.e.* cyclin D1, cyclin D3, CDK2, CDK4 and cyclin-dependent kinase inhibitors (p21 and p27), which are involved in G1 phase progression of the cell cycle [18–22]. Levomepromazine and buclizine significantly decreased cyclin D1, cyclin D3, CDK2 and CDK4 expression after 72 h (Figure 10). Changes of the cyclin-dependent kinase inhibitors, p21 and p27, were not observed (Figure 10).

### Western blot analysis of MCL-1L/S expression after treatment with levomepromazine or buclizine

We tested the effect of levomepromazine and buclizine on the expression of the anti-apoptotic MCL-1 and its pro-apoptotic variant, MCL-1S, in MCF-7 cells by Western blot (Figure 10).

**Figure 11:**
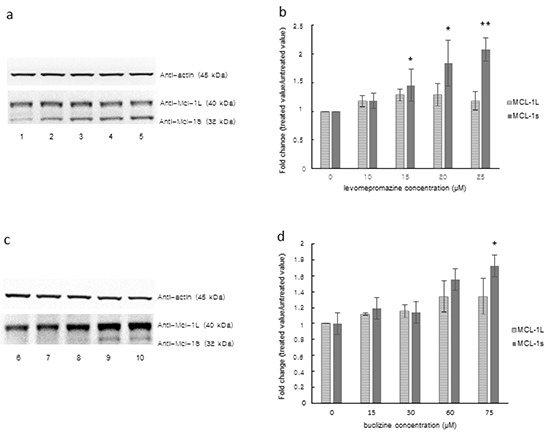
MCL-1L and MCL-1S expression after treatment with levomepromazine **a, b. or buclizine c, d. for 72 h as analyzed by Western blot,** Lane 1: solvent (H_2_O), Lane 2: 10 μM, Lane 3: 15 μM, Lane 4: 20 μM, Lane 5: 25 μM, Lane 6: solvent (DMSO), Lane 7: 15 μM, Lane 8: 30 μM, Lane 9: 60 μM and Lane 10: 75 μM (Significantly different according to Student's *t*-test, * 0.01 < *P* ≤ 0.05, **P ≤ 0.01), Quantification of MCL-1L and MCL-1S (b and d) expression by ImageJ. Western blots were performed three times.

Levomepromazine slightly increased MCL-1S expression, however, without reaching statistical significance (Figure 11a and 11b). Buclizine increased pro-apoptotic MCL-1S expression, while MCL-1L expression was slightly increased (Figure 11c and 11d).

### Trypan blue exclusion test of cell viability

**Figure 12:**
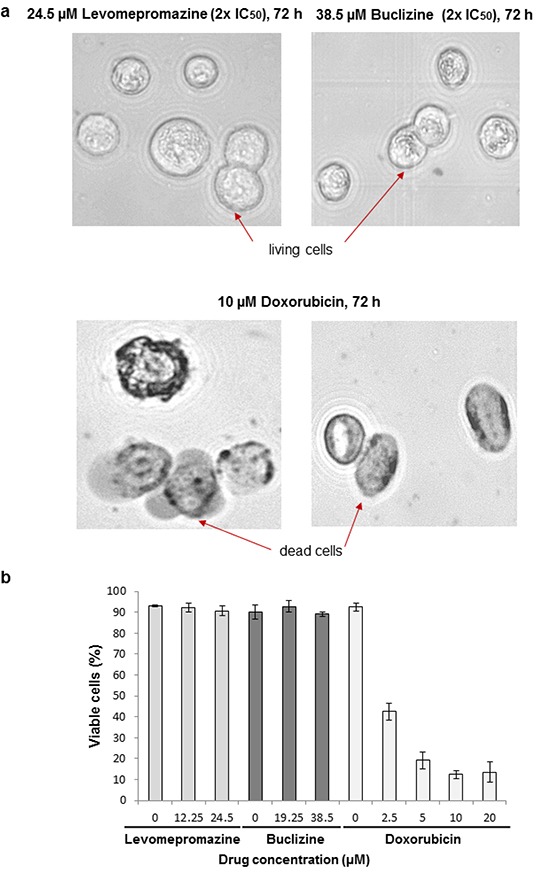
Analysis of cell viability by trypan blue staining of MCF-7 cells treated with IC_50_ or 2 × IC_50_ concentrations of levomepromazine or buclizine as well as 10 μM or 20 μM doxorubicin for 72 h **a.** Representative photographs (80× magnified transmitted light microscope) **b.** The graph of mean values ± SD of three independent experiments are shown.

We examined cell viability after treatment of MCF-7 cells with IC_50_ or 2 × IC_50_ of levomepromazine or buclizine for 72 h. More than 90% cells were viable after treatment with these two drugs (Figure 12). Doxorubicin is well known as a cytotoxic anticancer drug [23] and was used as control drug, the number of viable cells was dramatically reduced after treatment with 2.5 μM, 5 μM, 10 μM or 20 μM doxorubicin (Figure 12)

### Induction of differentiation as determined by lipid droplets staining

**Figure 13:**
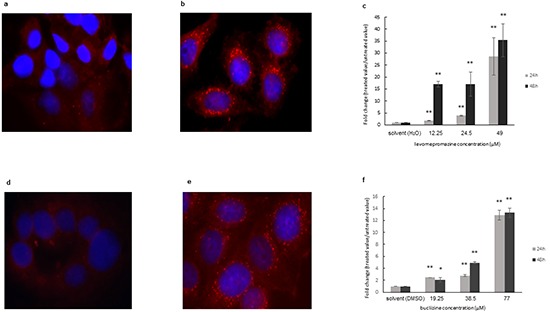
Induction of cellular differentiation after treatment of MCF-7 cells with levomerpomazine a-c. or buclizine d-f. for 24 h and 48h as detected by Nile Red staining a: solvent (H_2_O), 24h, b: 49 μM, 24h, d: solvent (DMSO), 24h, e: 77 μM, 24h, (Significantly different according to Student's *t*-test,* 0.01 < *P* ≤ 0.05, **P ≤ 0.01), Quantification analysis of lipid droplets (c and f) expression by ImageJ.

As the compounds induced cell cycle arrest, but not or only minimal apoptosis, we hypothesized that the observed inhibition of proliferation may be due to the induction of cellular differentiation, rather than to cytotoxicity. Therefore, we tested the formation of lipid droplets as a marker of breast cancer cell differentiation [24, 25]. Staining with the fluorescent dye Nile Red showed that solvents-only treated MCF-7 cells contained only few lipid vacuoles (Figure 13a and 13d). Lipid droplets significantly increased after levomepromazine (Figure 13b and 13c) or buclizine treatment (Figure 13e and 13f) for 24 h or 48 h treatment.

## DISCUSSION

We investigated the interaction of TCTP and antihistamic compounds to device new strategies for cancer therapy. TCTP is ubiquitously expressed in all eukaryotic organisms and in more than 500 tissues and cell types investigated [26]. TCTP expression levels are much higher in tumors compared to their corresponding normal tissues [16, 27]. One of the most convincing arguments speaking for a causative connection of TCTP to cancer biology is that the suppression of TCTP expression resulted in a reversion of the malignant phenotype [16]. TCTP plays an important role in cancer biology and participates in various cellular processes including protein synthesis, cell survival and cell growth [15]. Furthermore, a high-throughput screening analysis for differentially expressed genes between parental tumor cells and their revertants revealed that TCTP showed the most noticeable down-regulation [11, 16]. Therefore, TCTP represents an attractive target for therapy.

We chose antihistamic compounds for our study as potential TCTP inhibitors, because TCTP also acts as histamine-releasing factor. Thus, antihistamic compounds might inhibit TCTP. Importantly, antihistaminics are widely used in cancer patients as antiallergic, antidepressive or antiemetic agents. Moreover, some phenotiazines, including promethazine, thioridazine, perphemazine and chlorpromazine revealed antiproliferative effects [28–30]. Besides, antihistaminic compounds decreased TCTP expression, kill cancer cells and, eventually, led to strong reversion of the malignant phenotype [9]. Tuynder et al. reported that hydroxyzine and promethazine inhibited cell growth on human leukemia U937 cells and decreased TCTP expression on breast cancer MDA-MB231 and monocytic leukemia U937 cell lines [9]. These two drugs were also investigated *in vivo.* The volumes of MDA-MB231 and U937 xenograft tumors were consistently reduced by treatment with hydroxyzine and promethazine, indicating that these drugs indeed inhibited tumor growth by targeting TCTP [9]. Therefore, we decided to perform a systematic investigation on histaminic drugs.

First, we investigated the binding of 12 antihistamic compounds on TCTP by *in silico* molecular docking studies. We selected promethazine and hydroxyzine as control drugs, because they are well-known antihistaminics, which exert cytotoxicity towards cancer cells [9]. Hydroxyzine belongs to the piperazines and promethazine is a phenotiazine. Both of them antagonize the H1 receptor [31]. Promethazine interferes with histaminic effects of endotoxins against solid tumors [31]. We found that levomepromazine and buclizine bound to the same sites at TCTP as promethazine and hydroxyzine, but with even higher affinities. These compounds bound to the calcium binding site of TCTP (residues of 1-70) described by Graidist et al. [32]. Glu 58 and Glu 60 of TCTP residues are critical for calcium binding [32]. We observed that Glu 60 of TCTP was involved in hydrophobic interaction to hydroxyzine and buclizine. However, no hydrogen bond between Glu 58 or Glu 60 of TCTP residues and the control drugs or selected compounds was predicted by our *in silico* molecular docking studies. Thus, we predicted that the two selected compounds bound to the calcium binding site of TCTP.

Furthermore, we experimentally confirmed the direct binding of levomepromazine and buclizine to human TCTP using microscale thermophoresis (MST). This technique measures the motion of molecules in microscopic temperature gradients. There are several methods to measure the affinity of interacting molecules such as surface plasmon resonance (SPR) and isothermal titration calorimetry (ITC) [33]. SPR detects electromagnetic surface waves on a thin metal film [34]. These fields are strongly enhanced in resonance and are sensitive to the dielectrical properties of the surface and adjacent layers of surface-coupled molecules and solvent [34]. However, the covalent coupling of a molecule to a surface can interfere with the binding event. SPR also suffers from artifacts stemming from mass transport limitations close to an interface (i.e. rebinding effects and concentration depletion) [35]. Isothermal titration calorimetry (ITC) is a label-free method and measures the dissipated or absorbed reaction heat [33, 36, 37]. It allows direct access to the thermodynamics of an interaction [33]. To obtain measurable amounts of heat, high concentrations of the binding partners are required [33]. In contrast, MST is a very sensitive method to measure the equilibrium affinity constants of interactions, because it detects changes in size and charge as well as changes in the hydration shell of a molecule [33]. Moreover, MST has a low sample consumption and measures interactions with essentially no limitation on molecule size or molecular weight [33]. Due to these advantages, we applied MST for our investigation to study the interaction between human TCTP and antihistaminics.

We further explored the effects of two selected compounds on cell growth and TCTP protein and found indeed that the two compounds inhibited cell growth and down-regulated TCTP expression in MCF-7 cells, suggesting TCTP binding and down-regulation as causative growth-inhibitory mechanism of levomepromazine and buclizine. We could already observe significant down-regulation of TCTP at a concentration of 10 μM levomepromazine, which is lower than the IC_50_ concentration (Figure 7). However, TCTP protein was slightly down-regulated with 15 μM, 30 μM of buclizine and significantly down-regulated with 60 μM of buclizine, a concentration which is higher than the IC_50_ value. For comparison, Tuynder et al. treated U937 cells with 3.75-fold IC_50_ concentration of sertraline (15 μM), which inhibited tumor growth and changed TCTP protein expression [9].

Moreover, we investigated the cell cycle distribution of MCF-7 cells after drug treatment by flow cytometry. The percentage of G1 phase cells after levomepromazine or buclizine treatment increased in a concentration-dependent manner (Figure 8). However, we did not see increased apoptosis (Figure 8). Thus, the net growth was inhibited by G1 arrest rather than by apoptosis. Moreover, we further investigated the mode of the action of levomepromazine and buclizine towards MCF-7 cells using annexin V-PI staining (Figure 9). IC_50_ or 2 × IC_50_ concentrations of both drugs for 72 h treatment did not increase the fraction of dead cells and most of cells were annexin V/PI-negative, indicating that they were still alive (Figure 9). This result indicates that these two drugs cause neither necrosis nor apoptosis. Thus, they are not cytotoxic. Therefore, our cell cycle analysis and annexin V-PI staining results strongly suggested that levomepromazine and buclizine caused cell growth inhibition by G1 cell cycle arrest without induction of cell death (Figure 8 and 9). Zhang et al. demonstrated that TCTP promoted binding of proteins to damaged DNA that are components of the non-homologous end-joining mode of DNA double-strand break repair in G1-phase [38]. TCTP regulated radiation-induced G1 arrest, which is involved in DNA damage-sensing and repair [38]. Our observation that G1 arrest was associated with TCTP down-regulation after treatment with these two drugs corresponds to these data [38].

In addition, we investigated the expression of cyclin/CDK proteins such as cyclin D1, cyclin D3, CDK2 and CDK4, which are involved in G1/S progression in mammalian cells [39]. CDKs require complex formation with appropriate cyclin proteins for activation as well as phosphorylation by CDK-activating kinase [39, 40]. Cyclin proteins are overexpressed in breast cancer cells [41, 42]. In our study, we observed a significant decrease of cyclin D1, cyclin D3, CDK2 and CDK4 expression in MCF-7 cells treated with the two antihistaminics (Figure 10). These results indicate that down-regulation of cyclin/CDK proteins in levomepromazine- or buclizine-treated MCF-7 cells may be closely related to G1 cell cycle arrest. CDK inhibitors negatively control CDK activity and act as tumor suppressors [43]. However, we did not see significant changes of CDK inhibitors, p21 and p27, in levomepromazine- or buclizine-treated MCF7 cells. These results indicate that p21 and p27 are not involved in levomepromazine- or buclizine-mediated inhibition of cell growth. Our results are similar to other studies showing that retinoic acid treatment caused G1 arrest, but did not increase CDK inhibitors on MCF-7 cells [44, 45].

MCL-1 (myeloid cell leukemia-1) is an anti-apoptotic Bcl-2 family member, which contains BCL-2 homology (BH) domains 1, 2 and 3 and a C-terminal transmembrane region [46, 47]. A short splicing variant of MCL-1, termed MCL-1 short (MCL-1S), has an altered C-terminus as compared with the full-length MCL-1 long (MCL-1L) and lost BH1, BH2, and the transmembrane domain [48]. Overexpression of MCL-1S induces apoptosis and represents a pro-apoptotic BH3 domain-only protein capable of dimerizing with and inhibiting the anti-apoptotic MCL-1L [48]. Interestingly, we found a dose-dependent increase of MCL-1S expression after treatment with levomepromazine and buclizine. Anti-apoptotic MCL-1L expression was not significantly changed, but slightly increased after treatment with the two antihistaminics. The fact that the two drugs reduced TCTP expression and increased pro-apoptotic MCL-1S expression without significant changes of anti-apoptotic MCL-1L indicates that TCTP activity is not tightly limited with the apoptotic machinery. We investigated the cell cycle distribution of MCF-7 cells after treatment with levomepromazine (0-49 μM) and buclizine (0-77 μM) by flow cytometry (Figure 8). The percentage of G1 phase cells after treatment with these two drugs increased in a concentration-dependent manner (Figure 8). However, we did not see increased apoptosis, even at concentrations higher than the IC_50_ values of levomepromazine and buclizine (Figure 8). Hence, these two drugs induced G1 phase arrest but not apoptosis. We also studied MCL-1 L/S expression in MCF-7 cells treated with 0-25 μM levomepromazine or 0-75 μM buclizine. Increased pro-apoptotic MCL-1S expression was observed only at high drug concentrations without significant changes of anti-apoptotic MCL-1L (Figure 11). We conclude that apoptotic cell death is not a major mode of action of these compounds.

TCTP is a multifunctional protein and the question is, whether the drugs inhibit all or only specific functions of this protein. TCTP interacts with the anti-apoptotic Mcl-1 [49, 50] and Bcl-xL [51]. TCTP stabilizes Mcl-1 through interfering with Mcl-1's degradation by the ubiquitin-dependent proteasome degradation pathway [49]. The p53 tumor suppressor is another TCTP-interacting protein [52], which arrests the cell cycle arrest in late G1 phase and induces of apoptosis [53]. Ca^2+^ also binds to TCTP, which is a crucial second messenger for many cellular processes [32]. Interestingly, Bcl-2 proteins also possess non-apoptotic roles, including G1 cell cycle arrest and Ca^2+^ may be involved in these processes [54].

Levomepromazine acts as an antagonist of histamine type 1, muscarinic-cholinergic, dopaminergic 2, alpha-1 adrenoceptor and 5HT-2 receptors [55, 56]. Due to its broad-spectrum action on receptors involved in emesis, levomepromazine is effective as first-line treatment for intractable patho-physiological conditions and as second-line option for the treatment of nausea and vomiting [57–62]. Most studies indicated that levomepromazine doses up to 30 mg (=91 μM) per day can be used [57–62]. The bioavailability of levomepromazine *per os* is 20-40% (up to 30 μM). The maximal serum concentrations are achieved within 1-3 h after oral administration [63, 64]. The excretion is slow with a half-life of 15-30 h [63].

Buclizine is an antiemetic/antivertigo drug derived from piperazine that has anticholinergic and antihistaminic properties. It is used to treat nausea, vomiting and dizziness of motion sickness [65]. Buclizine can be used up to 75 mg (173.2 μM) per day without significant abnormalities in blood or in hepatic or renal functions [66, 67]. The peak plasma concentration of buclizine was 0.45 ± 0.10 μg/mL (8.08 μM -12.7 μM) and time to reach the peak plasma concentration was 3 ± 0.50 h with a half-life of 5.3 ± 3.01 h [68].

In our study, 0-25 μM levomepromazine or 0-75 μM buclizine were used to investigate the effects on TCTP, cell cycle regulatory proteins and MCL-1L/S expression in MCF-7 cells. These concentrations comprise the range of achievable clinical concentrations. The IC_50_ value of levomepromazine was 12.21 μM, which represents a concentration reachable in blood plasma [63, 64]. However, the IC_50_ value of buclizine was 19.18 μM and it is higher than maximal buclizine concentration in plasma [68]. Further investigations are warranted to clarify, whether buclizine derivatives with improved binding affinities might be developed to overcome this obstacle.

Trypan blue exclusion test was applied to investigate the number of viable cells after treatment with our two drugs (Figure 12). We confirmed that more than 90% of cells are living cells possessing intact cell membranes that excluded trypan blue staining after treatment with IC_50_ or 2 × IC_50_ concentrations of levomepromazine or buclizine for 72 h (Figure 12). This result is another evidence that these two drugs inhibited cell growth without inducing cell death. Therefore, we conclude that the IC_50_ values obtained by resazurin assays were due to cell growth inhibition. The cells could not be divided properly, and subsequently, the number of cells was dramatically reduced (Figure 6).

Based on our results, we conclude that the interaction of TCTP with the apoptotic machinery is not of major relevance for the anti-proliferative effects of antihistaminic compounds. The effect of these drugs on cell cycle arrest (Figure 8), Annexin V-PI staining analysis (Figure 9), cell cycle-regulating proteins (Figure 10) and cell viability using Trypan blue staining (Figure 12) confirm that cytostatic rather than cytotoxic mechanisms may be operative.

Cancer cells fail to differentiate into functional mature cells and differentiation therapy aims to re-inducing differentiation backwards to the non-malignant cellular states. This process is termed tumor reversion [69]. TCTP is down-regulated in tumor reversion and reduced TCTP levels were indeed responsible for reprogramming of cancer cells into revertants that lost most of their malignant phenotype [9, 16]. In addition, TCTP induction correlated with the mitogenic activity of non-neoplastic and cancer cell lines, indicating an involvement of TCTP in proliferation- and differentiation-related processes [13, 70].

Antihistaminics are widely used in cancer patients as antiallergic, antidepressive or antiemetic agents. However, their use as anticancer agents is clinically not established [9]. Histamine exerts its diverse effects through four receptors. Through the H_1_ receptor, histamine is involved in cell proliferation and differentiation [71]. It was reported that promethazine, sertraline and thioridazine delayed tumor formation [9]. Meclizine belongs to peperazine-type antihistaminics and is an antagonist at H_1_ receptors. It facilitated differentiation of chondrocytes by attenuating abnormally activated FGFR3 signaling in achondroplasia [72]. Levomepromazine and buclizine are H_1_ receptor antagonists. Therefore, we hypothesized that these compounds might be involved in cellular differentiation.

To confirm that two drugs really induced differentiation, we performed lipid droplets staining. Lipid droplets are a reliable marker for functional differentiation of mammary tissues [24]. They are mainly composed of triglycerides and contain important components of milk [24]. Nile Red is a vital dye that stains the components of intracellular lipid droplets [24]. We demonstrated that two antihistaminics indeed induced differentiation in MCF-7 cells by increase of lipid droplets (Figure 13).

Differentiation therapy aims to halt cancer growth by inducing cell differentiation [73]. This approach is based on the assumption that specific neoplastic cells exhibit aberrant patterns of differentiation and that treatment with appropriate agents results in tumor reprogramming, ultimately leading to a loss in proliferative capacity and induction of differentiation [73]. Conventional chemotherapy is frequently associated with the development of drug resistance and high toxicity, both of which limit its therapeutic efficacy [56]. A novel and potentially less toxic form of cancer therapy comprise agents that modify the state of differentiation and growth of cancer cells [73]. Clinically, all-trans retinoic acid (ATRA) is successfully applied for acute promyelocytic leukemia (APL) with an aberrant chromosomal translocation [74]. This translocation results from the fusion of the *PML* gene with the RA receptor gene (*PML-RARα*)[69]. ATRA differentiates APL cells into mature neutrophils [75, 76]. Another differentiating agent is sodium phenylbutyrate, which is FDA-approved to treat patients with hyperammonemia [69]. It inhibits histone deacetylase (HDAC) and exerts cellular differentiation through modification of chromatin and reprogramming of gene expression [77]. In addition, vitamin D3 has been shown to induce maturation of HL-60 and U937 leukemia cells [78, 79]. Vitamin D3 induced of CDKIs such as p27^KIP1^ and perturbated in the subcellular distribution of protein phosphatases [80, 81]. Besides, vitamin D3 synergistically interacted with other differentiation inducers (*e.g.* phorbol 12-myristate 13-acetate (PMA)) to trigger maturation of leukemia cells [81].

TCTP represents an exquisite target for differentiation therapy, since down-regulation of TCTP was indeed responsible for the reprogramming of cancer cells into revertants. Tuynder et al. showed that promethazine and hydroxyzine inhibited TCTP [9, 16]. Drugs targeting TCTP for differentiation therapy are not explored in detail as of yet and TCTP-based cancer therapy is still in its infancy. In the present study, we systematically investigated antihistaminic drugs. Levomepromazine and buclizine downregulated TCTP expression and inhibit cancer cell growth by direct binding to TCTP. Moreover, inhibition of TCTP by levomepromazine and buclizine affected specifically only the G1 phase cell cycle arrest without apoptosis in MCF-7 cells. Therefore, these two drugs did not cause cytotoxic effects although they inhibited proliferation. These compounds are valuable lead compounds for the generation of derivatives improved binding affinities to TCTP. It can be envisioned that differentiation therapy with higher tumor specificity and less side effects than cytotoxic tumor therapy can be reached with antihistaminic TCTP inhibitors.

## MATERIALS AND METHODS

### Molecular docking

The human TCTP structure was retrieved from PDB database (PDB code: 2HR9). PubChem was referred for the 3D structures of 12 antihistaminic drugs (https://pubchem.ncbi.nlm.nih.gov/). Molecular blind and defined docking calculations were performed with AutoDock4 [82]. The protocol for molecular docking was published by us [83].

For blind docking, Grid maps were created covering whole residues and number of energy evaluations was set to 25,000,000 and number of runs was set to 100 for the blind dockings. For defined docking, detected residues involved hydrogen bond or hydrophobic interaction with each ligand by blind docking were selected for grid maps. Docking parameters of defined dockings were set to 250 runs and 2,500,000 energy evaluations each time. Lamarckian Genetic Algorithm was chosen for docking calculations. For the visualization of docking results, AutodockTools-1.5.7rc1 was used. The surface representation image showing the binding pocket of human TCTP was made with Visual Molecular Dynamics (VMD) software developed with NIH support by the Theoretical and Computational Biophysics group at the Beckman Institute, University of Illinois at Urbana-Champaign (http://www.ks.uiuc.edu/Research/vmd/).

### Bacterial strains

*E. coli* K12 JM109 (F' traD36 proA^+^B^+^lacI^q^Δ(lacZ)M15/Δ(lac-proAB) glnV44 e14^−^ gyrA96 recA1 relA1 endA1 thi hsdR17) was used as host organism for gene manipulation. *E. coli* K12 ER2566 (F-λ-fhuA2 [lon] ompTlacZ:: T7 gene 1 gal sulA11 Δ(mcrC-mrr)114:: IS10 R(mcr-73:: miniTn10-Tet^S^)2 R(zgb-210:: Tn10)(Tet^S^) endA1 [dcm]) was chosen for heterogeneous gene expression. All strains used and/or constructed in this study are shown in Table 3.

**Table 3:** Strains used/constructed in this study

Strains	Characteristics	Sources
JM109	F' traD36 proA^+^B^+^lacI^q^Δ(lacZ)M15/Δ(lac-proAB) glnV44 e14^−^ gyrA96 recA1 relA1 endA1 thi hsdR17	NEB
JMTXB100	Ap^R^, JM109 harboring plasmid pTXB1	This study
JMTXB101	Ap^R^, JM109 harboring plasmid pTCTP01	This study
ER2566	F-λ-fhuA2 [lon] ompTlacZ::T7 gene 1 gal sulA11 Δ(mcrC-mrr)114::IS10 R(mcr-73::miniTn10-Tet^S^)2 R(zgb-210::Tn10)(Tet^S^) endA1[dcm]	NEB
ERTXB100	Ap^R^, ER2566 harboring plasmid pTXB1	This study
ERTXB101	Ap^R^, ER2566 harboring plasmid pTCTP01	This study

### Plasmids and enzymes

Plasmid vector pTXB1 (NEB, Ipswich, Massachusetts, USA) was used for cloning and expression of the target human *TCTP* gene. pTXB1 contains a mini-intein from the *Mycobacterium xenopi gyrA* gene (Mxe GyrA intein; 198 amino acid residues) that has been modified to undergo thiol-induced cleavage at its N-terminus.

All restriction enzymes and T4 DNA ligase were purchase from New England BioLabs (NEB, Ipswich, Massachusetts, USA) or Thermo Scientific (St. Leon-Rot, Germany).

### Design of human *TCTP* gene with optimized codon usage for expression in *Escherichia coli*

The sequence of the human *TCTP* gene (GenBank accession no. X16064.1) was optimized for high expression in *E. coli* according to the codon usage table (http://www.kazusa.or.jp/codon/). Optimized human *seTCTP* gene was synthesized by Eurofins MWG Operon (Ebersberg, Germany) as plasmid pEX-A-optimized human TCTP.

### Construction of recombinant plasmid encoding human *TCTP*

Plasmids used and generated in this study are listed in Table 4. In order to construct plasmid pTCTP01 encoding human *TCTP*, plasmid pEX-A-optimized human *TCTP* (optimized human *TCTP* gene, which was synthesized by Eurofins MWG Operon, Ebersberg, Germany) was digested with NdeI and SapI, optimized human *TCTP,* human *seTCTP,* gene (516 bp) was monitored in the 1% agarose gels. The desired DNA fragment was eluted using Gel Extraction Kit (Qiagen, Hilden, Germany). Subsequently, the eluted DNA was ligated with NdeI/SapI-cut pTXB1. *E. coli* K12 JM109 was transformed with pTCTP01 by electroporation. The colonies obtained were checked for containing the proper insert with DNA sequencing analysis. *E. coli* K12 ER2566 strain was subsequently transformed with this recombinant plasmid.

**Table 4:** Plasmid used/constructed in this study

Plasmids	Characteristics	Sources
pTXB1	Ap^R^, contains a mini-intein from the *Mycobacterium xenopigyrA* gene (Mxe GyrA intein; 198 amino acid residues) that has been modified to undergo thiol-induced cleavage at its N-terminus.	NEB
pTCTP01	Ap^R^, pTXB1 containing human *seTCTP* gene under the control of the T7 promoter and encoding human TCTP-intein fusion protein with an C-terminal Intein-tag.	This study

### Expression of recombinant human TCTP in *Escherichia coli*

Five microliters of glycerol culture of the recombinant strain were added to 5 mL of LB medium (Tryptone, yeast extract, NaCl), supplemented with ampicillin (100 μg/mL). Cultures were grown at 37°C for 16 h. Subsequently, 5 mL of culture were used to inoculate 500 mL LB medium containing ampicillin as stated above. The cultures were incubated at 37°C and expression was induced at an OD_600_ of 0.5 with IPTG (final concentration 0.1 mM) at 37°C, 27°C or 18°C for various times.

### SDS-PAGE analysis

Total proteins from whole cells were prepared by centrifugation of induced cultures at 10,000 × g for 5 min and cells were resuspended in sterile distilled H_2_O. 6× Laemmli buffer (12% SDS, 0.06% bromophenol blue, 47% glycerol, 0.06 M Tris-HCl, pH 6.8) was added at an 1/6 volume of each sample and then samples were boiled at 99°C for 10 min. SDS-PAGE (10% acrylamide) was used to check the expression of the recombinant human TCTP protein under reducing conditions. Three microliters of Page Ruler Prestained Protein Ladder (10-170 kDa) (Thermo Scientific) were used as standard markers.

### Isolation of a soluble human TCTP by chitin-affinity chromatography with a C-terminal intein-tag (human TCTP-intein)

The induced cells were collected by centrifugation at 5,000 × g for 30 min. The cells were lysed in cell lysis buffer (20 mM Tris-HCl, pH 8.5, 500 mM NaCl) by sonication (15 sec bursts, 10 times, with a 15 sec cooling period between each burst). Lysed extract was separated from cell debris by centrifugation at 10,000 × g for 30 min, and then the supernatant was filtered (0.2 μm). The filtrate contained the soluble proteins. To yield the insoluble proteins, the pellet was further dissolved in 8 M urea. The respective human TCTP can be enriched by chitin-affinity chromatography due to an intein-tag. Chitin beads (NEB, Ipswich, Massachusetts, USA) were used for the affinity chromatographic purification of human TCTP. A chitin column (Qiagen, Germantown, MD, USA) was equilibrated with 12.5 mL of column buffer (20 mM Tris-HCl, 500 mM NaCl, 1 mM EDTA). The soluble fraction of cellular proteins was subjected to chitin-affinity chromatography. Afterwards, the column was washed with 300 mL of washing buffer (20 mM Tris-HCl, 500 mM NaCl, 0.1% Triton X-100, 1 mM EDTA), in order to reduce non-specific binding of *E. coli* proteins. To release the target protein, on-column cleavage was induced by the cleavage buffer (20 mM Tris-HCl, 500 mM NaCl, 1 mM EDTA, 50 mM DTT). After the quick flush, the column flow stopped and was incubated at 23°C for 24 h. Afterwards, the target protein was eluted from the column using 2 mL of the column buffer. Desalination and buffer exchange were performed by Amicon Ultra-15 10K (Millipore, Darmstadt, Germany).

### Microscale thermophoresis

Protein interaction studies between human TCTP and two ligands, buclizine and levomepromazine were performed using microscale thermophoresis as described [33, 84]. Protein was labeled according to the Monolith™ NT.115 Protein Labeling Kit BLUE-NHS (Amine reactive) (NanoTemper Technologies GmbH, Munich, Germany). The labeled human TCTP was titrated with buclizine or levomepromazine. As control, the binding of only human TCTP was titrated alone in assay buffer (50 mM Tris buffer pH 7.6 containing 150 mM NaCl, 10 mM MgCl_2_ and 0.05% Tween-20) or adapted assay buffer (50 mM Tris buffer pH 7.6 containing 150 mM NaCl, 10 mM MgCl_2_, 2% chloroform and 0.05% Tween-20) without ligands. Sixty-six nanomol of human TCTP was used. The final concentrations of levomepromazine were 350, 175, 87.5, 43.8, 21.9, 10.3, 5.47, 2.73, 1.37, 0.68, 0.34, 0.17, 0.085, 0.042, 0.021 and 0.011 μM in assay buffer. The concentrations of buclizine were 600, 300, 150, 75, 37.5, 18.75, 9.375, 4.688, 2.344, 1.171, 0.586, 0.293, 0.147, 0.073, 0.037 and 0.018 μM in adapted assay buffer including 2% chloroform. Experiments were performed using standard capillaries in the NanoTemper Monolith™ NT (NanoTemper Technologies GmbH, Munich, Germany) for blue dye fluorescence in buffer. All experiments were performed at three times.

### Resazurin reduction assay

Resazurin reduction assay was used to investigate the effect of growth inhibition of levomepromazine and buclizine towards MCF-7 cells. The assay is based on reduction of the indicator dye, resazurin, to the highly fluorescent resazurin by viable cells. Non-viable cells rapidly lose the metabolic capacity to reduce resazurin and, thus, do not produce fluorescent signal. Briefly, adherent cells were detached by 0.25% trypsin/EDTA (Invitrogen, Darmstadt, Germany) and 5,000 cells were placed in each well of a 96-well cell culture plate (Thermo Scientific) in a total volume of 200 μL. Cells were attached overnight and then were treated with different concentrations of drugs. After 72 h incubation, 20 μL resazurin (Sigma-Aldrich, Taufkirchen, Germany) 0.01% w/v in ddH_2_O was added to each well and the plates were incubated at 37°C for 4 h. Fluorescence was measured by an Infinite M2000 Proplate reader (Tecan, Crailsheim, Germany) using an excitation wavelength of 544 nm and an emission wavelength of 590 nm. Each experiment was done at least three times, with six replicates each. The viability was analyzed based on a comparison with untreated cells. IC_50_ values indicate the drug concentrations required to inhibit 50% of cell proliferation and were calculated from a calibration curve by linear regression using Microsoft Excel.

### Protein extraction and Western blot

Ten milliliters of M-PER^®^ Mammalian Protein Extraction Reagent (Thermo Scientific) were used after adding one tablet of Complete Mini Protease Inhibitor (Roche, Mannheim, Germany). MCF-7 cells were incubated at 6 well plates with treatment of levomepromazine or buclizine for 72 h. Afterwards, medium including drug or solvent was removed and 100 μL of prepared M-PER^®^ Mammalian Protein Extraction Reagent (Thermo Scientific) including protease inhibitor were added to each plate. The plates were inverted for 5 min, then cells were harvested using a cell scraper and transferred to a 1.5 mL tube. The cell solution was shaked for 30 min at 4°C. After centrifugation (14000 × g, 15 min, 4°C), the supernatant was obtained and the protein concentration was determined using NanoDrop1000 (Thermo Scientific).

Afterwards, SDS-PAGE was performed as mentioned above. After electrophoresis, proteins were transferred to PVDF membrane (Carl Roth, Karlsruhe, Germany) in Western blot buffer (Transfer buffer: 25 mM Tris, 192 mM Glycine, 20% Methanol, pH 8.0) with a tank blot blotting apparatus at 250 mA for 90 min. The membrane was blocked in 5% bovine serum albumin (BSA)/Tris-buffered saline-Tween 20 (TBS-T) for 1 h and probed with a corresponding antibody. All antibodies were purchased from Cell Signaling (Beverly, Massachusetts, USA). Anti-TCTP rabbit monoclonal antibody (1:1,000), anti-Mcl-1 rabbit monoclonal antibody (1:1,000), anti-CDK2 rabbit monoclonal antibody (1:1,000), anti-CDK4 rabbit monoclonal antibody (1:1,000), anti-cyclin D1 rabbit monoclonal antibody (1:1,000), anti-cyclin D3 mouse monoclonal antibody (1:2,000), anti-p21 Waf1/Cip1 rabbit monoclonal antibody (1:1,000), anti-p27 Kip1 rabbit monoclonal antibody (1:1,000), anti-rabbit antibody (HRP-conjugated IgG; 1:2,000) and anti-mouse antibody (HRP-conjugated IgG; 1:2,000) were used in this work. The blots were processed for chemi-luminescence detection (Luminata™Classico Western HRP substrate, Millipore, Darmstadt, Germany).

### Cell cycle analysis by flow cytometry

For cell cycle analysis, MCF-7 cells were treated with various concentrations of buclizine or levomepromazine for 72 h. Afterwards, cells were washed by PBS and fixed in ice-cold 95% ethanol (Sigma-Aldrich, Germany). Fixed cells were washed with PBS and were incubate with 50 μg/mL propidium iodide (PI, Carl-Roth) in PBS for 1 h in the dark. Cells were measured by BD FACS Calibur flow cytometer (Becton-Dickinson, Heidelberg, Germany). 1 × 10^4^ cells were counted for each sample. PI was measured with 488 nm excitation (100 mW) and detected using a 610/20 nm band pass filter. Cytographs were analyzed using FlowJo software (Celeza, Olten, Switzerland). All experiments were performed three times.

### Assessment of cell growth inhibition by annexin V-PI staining

Cells were treated with IC_50_ and 2 × IC_50_ of levomepromazine and buclizine or with 10 μM and 20 μM doxorubicin for 72 h. Afterwards, cells were analyzed by annexin V-PI double staining (Bio Vision, Heidelberg, Germany). Annexin V is an intracellular protein that calcium-dependently binds to phosphatidylserine (PS), which translocates from the intracellular leaflet of the plasma membrane to the external leaflet during early apoptosis. Propidium iodide (PI) is excluded by living or early apoptotic cells with intact membranes and stains late apoptotic and necrotic cells with red fluorescence due to DNA intercalation. Therefore, cells with annexin V (-) and PI (-) are considered to be alive, while cells with annexin V (+) and PI (-) are in early apoptosis. Cells in late apoptosis or necrosis are both annexin V and PI positive. Briefly, MCF-7 cells were treated with various concentrations of levomepromazine, buclizine or doxorubicin for 72 h. After incubation, cells were collected by trypsination and centrifugation. After washing with medium, cells were incubated with annexin V and PI binding buffer (Bio Vision) according to the manufacturer's protocol. Subsequently, 2 × 10^4^ cells were counted and measured with Accuri™ C6 cytometer (BD Biosciences, Heidelberg, Germany). The annexin V-FITC signal was measured with 488 nm excitation and detected using a 530/30 nm band pass filter. The PI signal was analyzed with 561 nm excitation and detected using a 610/20 nm band pass filter. All parameters were plotted on a logarithmic scale. Cytographs were analyzed using BD Accuri C6 software (BD Biosciences).

### Trypan blue exclusion test of cell viability

A dye exclusion test was used to determine the number of viable cells present in a cell suspension [85]. It is based on the principle that live cells possess intact cell membrane that exclude certain dyes such as trypan blue. In this test, a cell suspension is mixed with dye and then visually examined to determine, whether cells take up or exclude the dye. Viable cell have clear cytoplasms, whereas non-viable cells appear with blue cytoplasms. Briefly, 5 × 10^5^ cells were centrifuged, and the cell pellet was resuspended in 1 mL of medium. Twenty microliters of cell suspension were mixed with 20 μL of 0.4% trypan blue. Twenty microliters of the trypan blue/cell mixture were applied to a hemacytometer and cells were observed by EVOS digital inverted microscope (Life technologies GmbH, Darmstadt, Germany). The unstained (viable) and stained (nonviable) cells were counted and the percentage of viable cell was calculated as following.

Viable cells (%) = total number of viable cells per ml / total number of cells per mL × 100.

### Lipid droplet staining for analysis of differentiation induction

MCF-7 cells were seeded in each well of a sterile ibi Treat *μ*-slide (ibidi, Martinsried, Germany) and cells were allowed to attach overnight. Cells were treated with different concentrations of levomepromazine, buclizine or solvent (H_2_O or DMSO). Afterwards, cells were rinsed twice with PBS and fixed for 20 min with PBS containing 4% paraformaldehyde at 20°C. After another PBS rinse and staining for 15 minutes with 500 nM of Nile Red (Sigma-Aldrich, Taufkirchen, Germany) and 300 nM of 4′,6-diamidino-2-phenylindole (DAPI) (Life Technologies GmbH, Darmstadt, Germany), the cells were PBS washed and mounted. Fluorescence imaging was performed by using 531 nm excitation and 593 nm emission for Nile Red and 357 nm excitation and 447 nm emission for DAPI of EVOS digital inverted microscope (Life Technologies).
